# Improving the quality of maternal and newborn health outcomes through a clinical mentorship program in the Democratic Republic of the Congo: study protocol

**DOI:** 10.1186/s12978-019-0796-4

**Published:** 2019-10-10

**Authors:** Xu Xiong, Rebecca Carter, Paul-Samson Lusamba-Dikassa, Elvis C. Kuburhanwa, Francine Kimanuka, Freddy Salumu, Guy Clarysse, Baudouin Kalume Tutu, Sylvain Yuma, Alain Mboko Iyeti, Julie H. Hernandez, Jeffrey G. Shaffer, Susie Villeneuve, Alain Prual, Lee Pyne-Mercier, Assaye Nigussie, Pierre Buekens

**Affiliations:** 1 Tulane University School of Public Health and Tropical Medicine, 1440 Canal St, Ste. 2000, New Orleans, LA 70112 USA; 2 Kinshasa School of Public Health, University of Kinshasa, Kinshasa, Democratic Republic of the Congo; 3 UNICEF, 372, avenue Colonel Mondjiba, Kinshasa-Ngaliema, Democratic Republic of the Congo; 4 Ministère de la Santé, Secrétariat général, 36 Avenue de la Justice, Kinshasa – Gombe, Democratic Republic of the Congo; 5 UNICEF Western & Central Africa Regional Office, PO Box 29720, Dakar-Yoff, Senegal; 6 Bill & Melinda Gates Foundation, PO Box 23350, Seattle, WA USA

**Keywords:** Clinical mentorship, Democratic Republic of the Congo, Emergency obstetric and neonatal care, Maternal and newborn health

## Abstract

**Background:**

The Democratic Republic of the Congo (DRC) boasts one of the highest rates of institutional deliveries in sub-Saharan Africa (80%), with eight out of every ten births also assisted by a skilled provider. However, the maternal and neonatal mortality are still among the highest in the world, which demonstrates the poor in-facility quality of maternal and newborn care. The objective of this ongoing project is to design, implement, and evaluate a clinical mentorship program in 72 health facilities in two rural provinces of Kwango and Kwilu, DRC.

**Methods:**

This is an ongoing quasi-experimental study. In the 72 facilities, 48 facilities were assigned to the group where the clinical mentorship program is being implemented (intervention group), and 24 facilities were assigned to the group where the clinical mentorship program is not being implemented (control group). The groups were selected and assigned based on administrative criteria, taking into account the number of deliveries in each facility, the coverage of health zones, accessibility, and ease of implementation of a clinical mentorship program. The main activities are organizing and training a national team of mentors (including senior midwives, obstetricians, and pediatricians) in clinical mentoring, deploying them to mentor all health providers (mentees) performing maternal and newborn health (MNH) services, and providing in-service training in routine and Emergency Obstetrical and Newborn Care (EmONC) to the mentees in health facilities over an 18-month period. Baseline and endline assessments are carried out to evaluate the effectiveness of the clinical mentorship program on the quality of MNH care and the effective coverage of key interventions to reduce maternal and neonatal mortality. Findings will be disseminated nationwide and internationally, as scientific evidence is scarce. A national strategy, guidelines, and tools for clinical mentorship in MNH will be developed for replication in other provinces, thus benefitting the entire country.

**Discussion:**

This is the largest project on clinical mentorship aimed to improving the quality of MNH care in Africa. This program is expected to generate one of the first pieces of scientific evidence on the effectiveness of a clinical mentorship program in MNH on a scientifically designed and sustainable model.

## Plain English summary

To improve quality of maternal and newborn care, the objective of this ongoing project is to design, implement, and evaluate a clinical mentorship program in 72 health facilities in two rural provinces in the DRC. In the 72 facilities, 48 facilities were assigned to the group where the clinical mentorship program is being implemented (intervention group) and 24 facilities were assigned to the group where the clinical mentorship program is not being implemented (control group). The groups were selected and assigned based on administrative criteria, taking into account the coverage of health zones, accessibility, and ease of implementation of a clinical mentorship program. The main activities include organizing and training a national team of mentors (including senior midwives, obstetricians, and pediatricians) in clinical mentoring, deploying them to mentor all health providers performing maternal and newborn health services (mentees), and providing in-service training in routine and Emergency Obstetrical and Newborn Care to the mentees in health facilities over an 18-month period. Baseline and endline assessments are ongoing to evaluate the effectiveness of the clinical mentorship program on quality of MNH care and effective coverage of key interventions to reduce maternal and neonatal mortality. This program is expected to generate one of the first pieces of scientific evidence on the effectiveness of clinical mentoring to improve maternal and neonatal health care and outcomes. If the program is effective, it will demonstrate the potential for the application of this model of a clinical mentorship program across the DRC and other sub-Saharan African countries.

## Background

Maternal and neonatal deaths are a global issue but disproportionately affect low- and middle-income countries (LMICs). An estimated 99% of maternal deaths occur in LMICs, and nearly two-thirds occur in the sub-Saharan African region [1]. There are an estimated three newborn deaths per 1000 live births in high-income countries compared to 27 newborn deaths per 1000 live births in low-income countries [1, 2]. Many maternal and newborn deaths are a result of preventable complications during pregnancy and childbirth or immediately after birth. Common causes of maternal death include severe bleeding, infections, hypertensive disorders in pregnancy, or delivery complications. Common causes of newborn death are premature birth, infections, or labor and delivery complications [2]. Several strategies are used to reduce maternal and neonatal deaths in LMICs, including improving quality and access to antepartum or antenatal care, increasing rates of institutional deliveries for better access to intrapartum and postpartum care, delivery assisted by a skilled birth attendant, and enhancing the structural capacity of facilities via the availability of life saving drugs and improved facility conditions [1, 2].

Quality of care, defined as “the extent to which health services provided to individuals and populations improve desired health outcomes,” is a result of many factors: quantity and quality of motivated human resources qualified in emergency obstetric and neonatal care (EmONC), the professional environment, and the availability of essential equipment, drugs, and supplies [3]. Provision of quality of health care and services delivered by appropriately prepared healthcare personnel is a crucial strategy aimed at reducing the high burden of diseases and deaths in LMICs [4–6]. However, LMICs often suffer from a shortage of primary healthcare providers, a lack of opportunity for continued education to improve provider performance (i.e., skills, knowledge, and attitudes), poor facility conditions, a lack of supplies, and compromised sociopolitical environments [7, 8]. One approach to improve the quality care is to address the lack of training and/or continued education through development and implementation of a clinical mentorship program. Clinical mentoring (CM) is the continued training and consultation of employees for professional development and improved health outcomes [9]. In 2006, the WHO published a report outlining the role of clinical mentoring in HIV care in resource constrained settings [9]. Since then, several studies have demonstrated the efficacy of clinical mentoring to improve quality of care in HIV/AIDs treatment in sub-Saharan African countries [10, 11]. However, few clinical mentorship programs have been developed and implemented to improve quality of maternal and neonatal care in LMICs [12–14].

The Democratic Republic of the Congo (DRC) boasts one of the highest rates of institutional deliveries in sub-Saharan Africa (80%) [15]. Additionally, eight out of every ten births is assisted by a skilled provider [15]. However, the maternal mortality ratio and neonatal mortality rates in the DRC are still among the highest in the world (846 maternal deaths/100,000 live births and 28 newborn deaths/1000 live births respectively) [2, 15], contributing to 4% of global newborn deaths [15, 16]. Taken together, this suggests that while the availability of healthcare workers is high, there may be obstacles regarding the quality of care provided. Therefore, there is a need for continued education for health care providers to improve their skills, knowledge, and attitudes on MNH care.

It is hypothesized that a clinical mentoring intervention program will lead to improvements in primary healthcare providers’ (e.g., mentees) technical skills, knowledge, attitudes, and clinical decision-making for better delivery of maternal and neonatal healthcare (MNH) services, thereby reducing maternal and neonatal morbidity and mortality [17–19]. The objective of this project is to develop, implement, and assess the effectiveness of a clinical mentoring program aimed to improve the quality of MNH care, as well as to reduce maternal and infant mortality rates in two provinces in the DRC.

## Methods

### Overview of study design

**Fig. 1:**
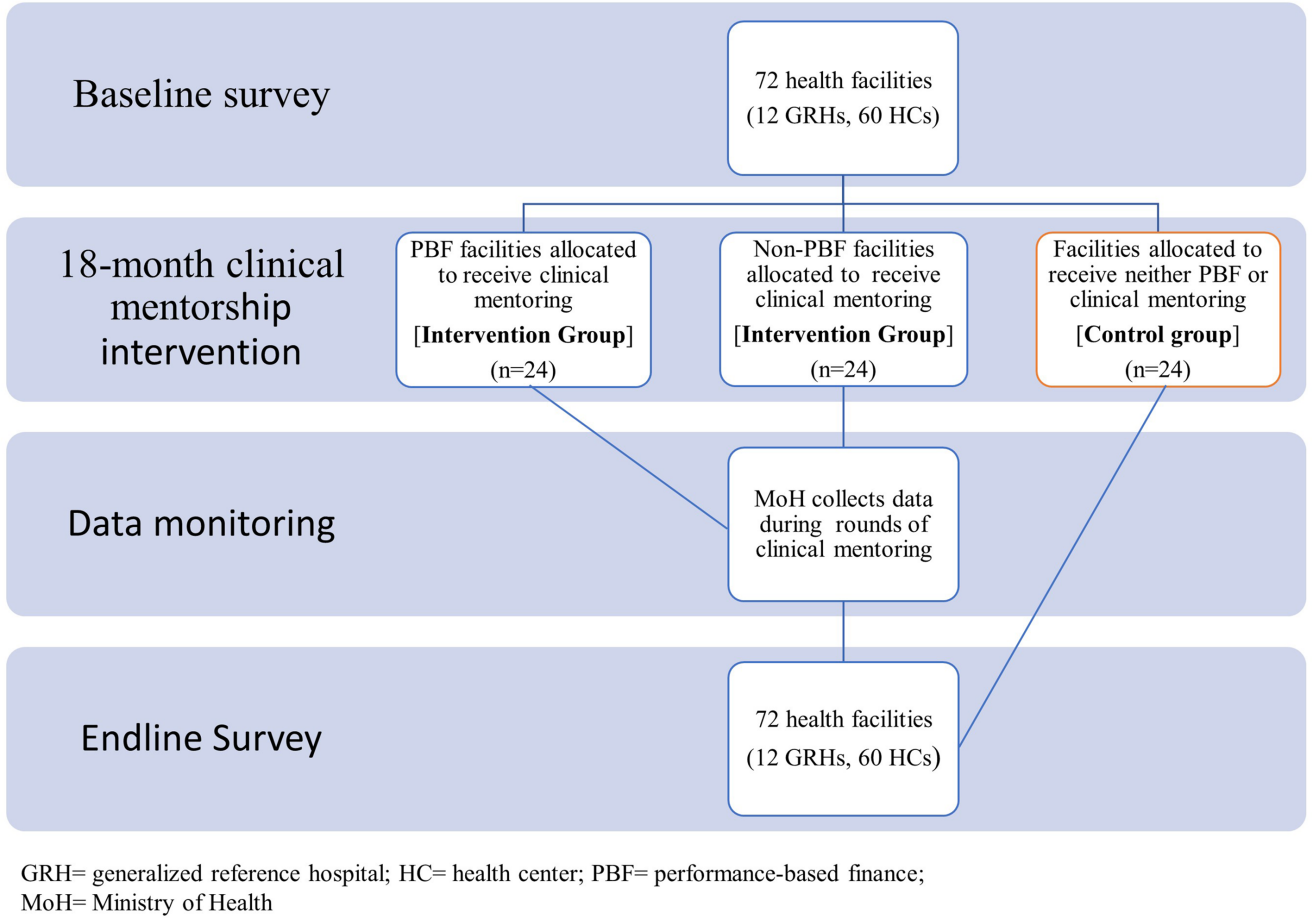
Overall study design

This is an ongoing, quasi-experimental study design. The project is currently being conducted in 72 health facilities (60 primary healthcare clinics and their 12 general referral hospitals) in the provinces of Kwango and Kwilu, DRC. Each province is comprised of health zones, and each health zone has at least one general reference hospital (GRH) and other health centers (HC). In these 72 facilities, 48 facilities were assigned to the clinical mentorship implementation group (i.e., intervention group), and 24 facilities were assigned to the group without clinical mentorship implementation (i.e., control group). Assignment of the two groups of health facilities was not performed randomly but according to administrative criteria. The intervention group is also balanced over the existing World Bank-funded Performance-Based Financing intervention (PBF) randomized control trial. Among the 48 health facilities where clinical mentorship is implemented, 24 were selected from PBF health zones and 24 were selected from non-PBF health zones (Fig. 1). This was done to limit the potential interference of the clinical mentorship program with the PBF project’s existing randomized trial. The primary outcomes are indicators of MNH care providers’ skills, knowledge, and attitudes. The secondary outcomes are maternal and infant outcomes (e.g., preeclampsia, forceps assisted delivery, cesarean-section, or blood transfusion, maternal deaths, infant deaths).

**Fig. 2:**
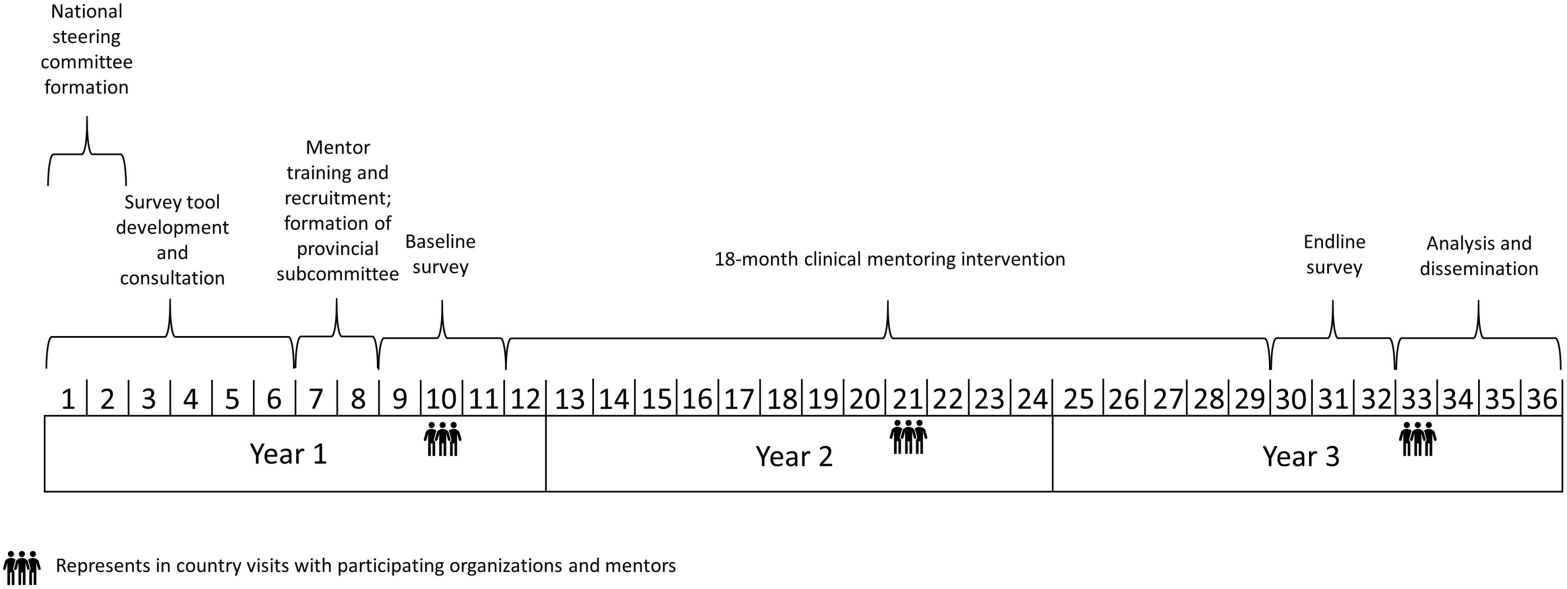
Timeline of study development. Each number corresponds to one calendar month and is representative of the 36 month timeline for the study development and implementation

The baseline survey collected information on primary and secondary outcomes, as well as information on facility conditions (e.g., equipment, medicine, structure) and an Emergency Obstetric and Newborn Care Needs Assessment (EmONC) at these 72 facilities. After completion of the baseline survey, we began implementation of an 18-month clinical mentorship period in the 48 intervention facilities. Process and outcome variables are being collected to assess the performance of the clinical mentors and mentees, as well as the clinical mentorship program. Upon completion of the clinical mentoring period, endline surveys will be conducted in the 72 facilities to collect the same primary and secondary outcomes and other variables. Effectiveness of the clinical mentorship program will be assessed through comparison of the primary and secondary outcomes between the baseline and endline surveys and between the intervention and control groups. The study requires 36 months (3 years) to complete. Figure 2 presents the timeline and summarizes key activities of the project.

**Fig. 3:**
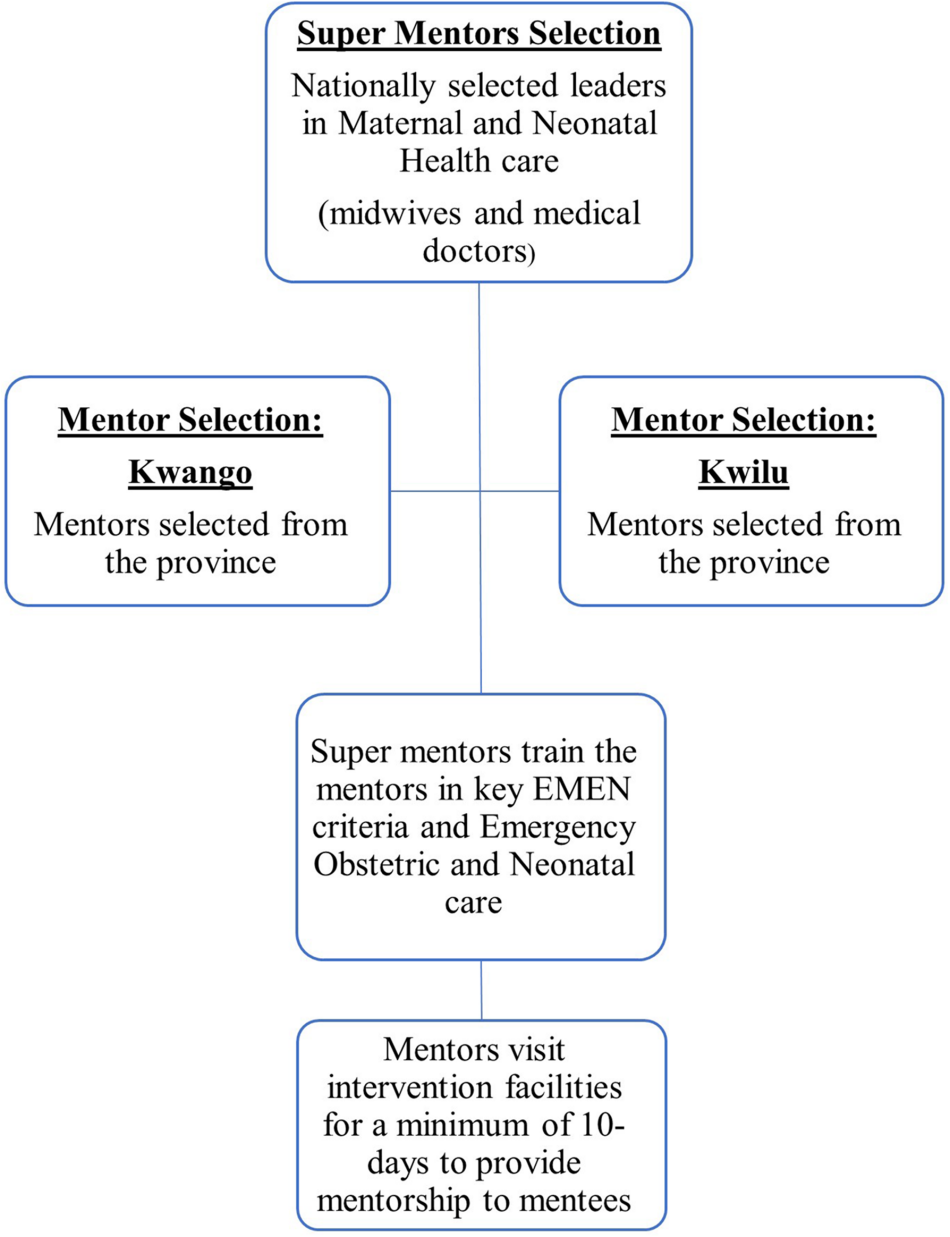
Schematic of clinical mentoring implementation

### Study setting and participants

In Kwango and Kwilu provinces, 72 health facilities from six health zones, including 12 general referral hospitals (GRHs) and 60 health centers (HCs), were selected. The selection and allocation of the health facilities were based on the following administrative criteria:

1. Size of health facility (at least 200 annual deliveries); this number of deliveries was selected to ensure that some adverse pregnancy complications and birth outcomes would occur during the total 6–8 weeks of clinical mentorship in each facility, thereby allowing the mentor to address some severe maternal and newborn conditions with the mentees.
2. Coverage of health zones (the majority of the health zones include at least one GRH and its affiliated HCs);
3. Accessibility (e.g., ability to reach selected health facilities with ease);
4. Ease of implementation of clinical mentorship program (e.g., selecting health facilities that are near one another to ease mentor travel).

All healthcare providers who provide MNH care and services in the 72 health facilities are eligible to participate as mentees in this clinical mentorship program. Written consent was obtained to collect data on their performance of routine MNH care [7].

### Clinical mentorship program

#### Development of clinical mentorship intervention

The WHO defines clinical mentoring (for HIV care and antiretroviral therapy) as “a system of practical training and consultation that fosters ongoing professional development to yield sustainable high-quality clinical care outcomes … [that is] critical to building successful district networks of trained health care workers [9].” The clinical mentorship program developed for this project combines the WHO model of quality of care and UNICEF’s Every Mother Every Newborn (EMEN) guide for quality improvement [20, 21]. The mentoring program was designed to meet two core principles. First, to continuously optimize performance of MNH services, given the resources at hand, through clinical mentorship in the context of the DRC. Second, to support the MNH services to deliver “safe, effective, timely, efficient, equitable, and people-centered services” [21]. The clinical mentorship program was designed to cover the EMEN 10 standards of care (see Panel: EMEN 10 criteria). These principles guided the development of the questionnaires used in the project’s monitoring and evaluation. The primary assessment was centered around the first three points: the provision of evidence-based practices during antenatal care, labor and childbirth, and postnatal care [21].

The clinical mentoring program was created in conjunction with an international consultant. Given that clinical mentorship for the improvement of MNH care is a new approach in the DRC, an orientation on quality improvement and clinical mentorship was organized for the key national and provincial stakeholders. Stakeholders included, but were not limited to, national and international partners, policy makers, program managers, and professional associations. A National Steering Committee was created under the umbrella of the national Maternal Newborn Child and Adolescent Health (MNCAH) Task Team. The committee meets quarterly, and a regional workshop was organized with experts in clinical mentoring to oversee, develop, and implement the clinical mentorship program. A provincial subcommittee participated in the design of the clinical mentoring program and assists with the coordinating and monitoring of the project. The teams of the “Provincial Division of Health” of the two targeted provinces and the “Health Zone Framework Teams” of the eight targeted health zones are oriented and receive key information on the project. Their role is to coordinate and supervise the mentors’ field activities during their routine supervision of the health facilities during the 18 months of the clinical mentorship implementation, with the support of UNICEF provincial offices.

#### Selection of mentors and mentees

UNICEF and MOH recruited a team of “super mentors” through a nationwide competitive process. Super mentors include currently practicing midwives, obstetricians, and pediatricians with extensive clinical experience in MNH care. The team of super mentors recruited highly skilled and actively practicing medical doctors and midwives to act as clinical mentors; they are now providing training and supervision of the clinical mentors throughout the project. The clinical mentors are experienced and skilled MNH care providers recruited from Kwango and Kwilu provinces. Their key responsibilities, while providing clinical mentorship, are to: (1) disseminate clinical practice guidelines and information to enhance patient outcomes; (2) assist in the ongoing training of mentees in the core clinical competencies in Emergency Obstetric and Neonatal Care; (3) integrate mentees’ clinical skills, knowledge, attitudes, and clinical decision-making; (4) provide effective feedback to mentees; and (5) determine if performance standards are being met. Figure 3 outlines mentor selection and implementation.

Mentees are health professionals performing MNH care in the selected GRH and HC facilities. Most of them have had little pre- and/or in-service training in the evidenced-based care previously mentioned. The general practitioners and nurses working in GRHs may have a stronger background, but they infrequently receive clinical support from professionals with advanced training like obstetricians and paediatricians. Therefore, continuing education through the clinical mentoring program to all consenting health professionals performing deliveries in the 48 intervention facilities is vital to evidence-based practice.

#### Implementation of clinical mentorship program

Among the team of clinical mentors, medical doctors provide mentorship in GRH facilities and midwives provide mentorship in HC facilities. At the intervention facilities, one mentor is responsible for visiting several facilities over the course of the 18-month clinical mentoring period. While visiting a facility, mentors remain at the facility for the duration of each clinical mentoring session. Mentors conduct a minimum of five mentoring sessions, each lasting at least 10-days, over the course of the 18-month intervention period, for a total of 6–8 weeks of clinical mentoring for each facility. Mentoring is begun after the baseline data collection is completed. At each facility, the clinical mentors are responsible for: (1) strengthening mentees’ knowledge, attitudes, and skills through observation and review; (2) assessing routine MNH practices; (3) guiding mentees through a re-examination of their ideas and values; and (4) ensuring the learning and personal/professional development of mentees. In addition to in-person mentoring, mentors are accessible by phone and web to provide advice or answer mentee’s patient care questions.

#### Monitoring and evaluation of implementation of clinical mentorship program

Before the start of the clinical mentoring program, selected mentors were trained in quality assurance and in clinical mentorship by the super mentors, who are national experts in the DRC. During the 18-month clinical mentorship implementation period, the performance of the clinical mentors and mentees is monitored and evaluated. The key monitoring questions are: (1) Is the implementation of the CM program being conducted as planned? (2) Are the clinical mentors performing according to the standards? (3) Are the mentees performing according to the expectations? (4) How is the clinical mentoring program impacting key MNH outcomes?

Table 1 is a summary of the instruments and data collection tools used in monitoring and evaluating the clinical mentorship program implementation. The DRC Ministry of Health Coordinator for each province is responsible for sending a monthly summary report of clinical mentors’ visits to the facilities, including the number of mentees completing the clinical mentoring sessions and the qualitative issues noted by the clinical mentor.

**Table 1:** A summary of the instruments and data collection tools for monitoring clinical mentorship program

Data collection tools	Frequency of data collection
**Instruments for Mentors Use**	
• Mentor’s self-report form	Per each mentoring session/facility
• Pre-mentoring mentee’s clinical skills, knowledge and attitude checklist	Per each mentoring session/facility
• Post-mentoring mentee’s clinical skills, knowledge and attitude checklist	Per each mentoring session/facility
• Mentee satisfaction	Per each mentoring session/facility
• Mentee’s log book	Per each mentoring session/facility
• 3-month delivery record review and abstraction^a^	Per each mentoring session/facility
**Instrument for DRC Ministry of Health Coordinator’s Use**	
• Monthly summary report	Monthly
**Instruments for Project Monitoring Team’s Use (for quality control)**	(For randomly selected facilities)
• Mentor’s activity monitoring form (by interviewing head of the facility)	Per facility
• Mentee’s logbook	Per facility
• Mentee’s satisfaction	Per facility
• 3-month delivery record review and abstraction^a^	Per facility
• Facility condition assessment^a^	Per facility

^a^the same tools used during baseline surveys [7]

### Data collection

Both the baseline and endline surveys collect data on the primary and secondary outcomes indicated above and facility conditions (equipment, medicine, structure). Data collectors use a pre-programmed, smartphone-based application, Open Data Kit [22] (ODK Collect v1.16), to complete the following checklists or questionnaires:

1. Facility condition form: This checklist includes an assessment of infrastructure, equipment, medicines & supplies, water supply & electricity, and staffing.
2. Health providers’ obstetric and neonatal care practice checklist: This checklist was created based on criteria from the Every Mother, Every Newborn (EMEN) Quality Improvement Guide for Health Facility Staff [21]. Final indicators were determined based on the feasibility to measure these indicators in the field. These indicators are the primary outcomes, measuring healthcare providers or mentees’ skills, knowledge, and attitudes during antenatal care, labor, delivery, and postnatal care [21].
3. A three-month delivery record review and extraction: A data extraction form was created to collect the secondary outcomes of key maternal and infant morbidity and mortality outcomes at the health facilities:
  - Maternal outcomes include number of deliveries, number of women with antenatal hemorrhage, pre-eclampsia/eclampsia, postpartum hemorrhage, frequency of cesarean section (if available), and maternal deaths.
  - Neonatal outcomes include the number of newborns with low birth weight (< 2.5 kg), preterm delivery, asphyxia, major infections, stillbirths, and neonatal deaths (< 7 days). When available, data on comprehensive EmONC measures is also collected (e.g., manual removal of the placenta or removal of residual retained products).

The baseline survey has been completed and published [7]. All data baseline and endline collection tools/questionnaires for the surveys are available for public access [23].

During each clinical mentoring session, mentors collect information through record review, direct observation, and patient chart review. Mentors use paper-based forms to collect the following information:

1. Mentor’s self-report form: This form outlines the total number of mentees who were available, consented, and completed a clinical mentoring session, with space to qualitatively note lack of participation or completion.
2. Mentees’ pre- and post- test knowledge assessment: Developed from the EMEN criteria, these identical forms are completed by mentors to assess mentees’ skills, knowledge, and attitudes during antenatal, labor, delivery, and postnatal care at the beginning and end of a clinical mentoring session.
3. Mentee satisfaction: This ten-item, Likert scale questionnaire is completed by mentees to assess their satisfaction with the clinical mentoring session.
4. Mentees’ logbook: This qualitative form outlines mentees’ patient interactions and care during the clinical mentoring session.
5. A three-month delivery record review and extraction: The same form that is used in baseline and endline surveys is used by mentors during the clinical mentoring intervention period.

### Sample size and statistical power calculation

The sample size including 72 health facilities (12 GRHs and 60 HCs) is an administrative decision taking into account increasing coverage of potential benefits of the clinical mentorship program to a large number of health facilities in the two participating provinces in this project. Statistical power was calculated to detect an increase of the key primary outcomes (e.g., proportion of monitoring progress of labor using partogram) or a decrease of a composite secondary outcome of severe newborn complications (e.g., low birth weight < 2.5 kg, preterm delivery, asphyxia, major infections, stillbirths, and neonatal deaths < 7 days). Based on this fixed sample size of 72 health facilities with at least 200 annual deliveries per each facility, under assumption of a significance level of the test of 0.05 and an intracluster correlation coefficient of 0.01, for example, the sample size provides more than 99% power to detect an increase of proportion of monitoring progress of labor with partogram from 50 to 75%, and a 30% reduction in the composite of severe newborn complications (from 15 to 10.5%).

### Quality assurance and control

Prior to the implementation of the project, data collectors were trained in how to use the ODK data collection system to complete the questionnaires. To ensure a high quality of data collection, a data collection coordination office was established in the capital city of Kikwit in Kwilu province. During the baseline and endline surveys, the data coordinator organizes and supervises six data collectors to visit all 72 facilities [7]; the data coordinator is also responsible for receiving the questionnaires/forms completed by mentors and the DRC Ministry of Health coordinator during the 18-month clinical mentorship implementation. For quality control purposes, the data coordinator conducts monitoring visits to randomly selected health facilities from the intervention facilities that have already received clinical mentoring to independently verify the data on mentors and mentees’ performance and to assess any changes of facility conditions (infrastructure, equipment, medicines & supplies, water supply & electricity, and staffing) (Table 1).

Hard copies of completed consent forms, questionnaires, or assessment forms are delivered to the Kikwit office, and these documents are analyzed by the Tulane University New Orleans team to ensure that the clinical mentoring program is being implemented as planned and to ensure the accuracy of data collected. Additional ongoing strategies for quality control include: 1) monthly conference calls among the project PI, Co-PIs, and key project personnel; 2) monitoring field visits to the participating health facilities and meetings/workshops with provincial project officers and coordinators, mentors, and selected mentees in the field; and 3) annual Steering Committee meetings, including all involved parties (UNICEF, Bill and Melinda Gates Foundation, Ministry of Health, national and provincial level officers, clinical mentors, and Tulane University) in the DRC.

### Data management

The ODK system is used for baseline and endline data collection. Data collected on smartphones is securely transmitted to a secure, cloud-based server (Google App Engin), and only key project personnel have access to the server. Hard copies of completed questionnaires/evaluation forms from clinical mentors are delivered to the data coordination office in Kikwit, and data is scanned and uploaded to a cloud-based archival location by the Kinshasa team. A Tulane University data center in New Orleans is responsible for receiving, managing, analyzing, and reporting the data after the baseline survey, on a quarterly basis during the implementation of the clinical mentorship program, and after the endline survey [7]. Data verification techniques include logic, range, and consistency checks. Data validation is implemented via electronic data entry mechanisms with input masks, conditional logic, and validation rules. Any issues are reported to all study coordinators and key personnel via weekly teleconferences.

### Data analysis plan

To examine if the clinical mentorship program leads to improvement in MNH indicators of providers’ skills, knowledge, and attitudes (primary outcomes), as well as provides for a reduction in maternal and neonatal morbidity and mortality (secondary outcomes), baseline and endline surveys are designed to compare changes in percentages of the primary and secondary outcomes before and after the implementation of the clinical mentoring program and between the intervention and control groups. To assess if the improvement of MNH quality of care and outcomes is due to the clinical mentorship program itself or due to PBF intervention, the indicators related to quality of care and maternal and newborn outcomes will be compared across three groups: 1) the 24 health facilities with both PBF and clinical mentorship interventions; 2) the 24 health facilities with clinical mentorship intervention only and without PBF intervention; and 3) the 24 health facilities both without PBF and clinical mentorship interventions (control group).

Chi-squared tests will be used to test differences in the distributions of categorical MNH indicators and outcome measures. Then a relative risk (RR) and 95% confidence interval will be calculated. The effectiveness assessment will be presented as either a percent increase in the process indicators (e.g., % of syphilis screening, woman receives uterotonic immediately after birth of the baby, or breastfeeding) or a percent decrease in maternal and newborn outcomes (e.g., maternal deaths, stillbirths, neonatal deaths< 7 days) that are attributable to the clinical mentorship program [i.e. Effectiveness = (1-RR) %] after adjustment of potential confounding factors.

Secondary, stratified analyses will be performed by health zone, type of health facilities (e.g., public vs. private or faith-based), GRH vs. HC, size or number of deliveries, 7- or 9-signal functions EmONC standards being met, type of providers (e.g., physicians, midwives, nurses), and performance of the clinical mentorship program (e.g., percentage of mentees completing the clinical mentorship program) to examine if the benefits of the clinical mentorship program are differentiated by these factors.

## Discussion

We are conducting an ongoing, quasi-experimental study to assess the effectiveness of a clinical mentorship program to improve providers’ skills, knowledge, and attitudes for delivering a higher quality of MNH care and reducing infant and maternal mortality rates in the Kwango and Kwilu provinces of the DRC. The aim of this program is to create a replicable model of MNH clinical mentorship that can be scaled to improve the quality of MNH care across the DRC and other sub-Saharan African countries. Clinical mentoring is an intervention that has previously been applied to the management of HIV/AIDS but with few applications to maternal and child health outcomes to date [12–14]. If this intervention is effective, it will be one of the first documented applications of clinical mentoring aimed to improve the quality of maternal and newborn health care and outcomes.

Clinical mentorship is a system of practical training and consultation that fosters ongoing professional development to yield sustainable, high-quality clinical care outcomes [9]. Poor MNH is not only a consequence of a lack of services but also the limited quality of care of existing services. Poor quality can also be due to a lack of resources, inadequate treatment, insufficient information exchange, and a lack of technical competency. Health professionals in a rural area of the DRC often work in isolation, in poor facility conditions with a lack of supplies, far from many resources, and with no available peers for advice and support. There is not a single model of clinical mentoring approaches that fits all settings [5, 6]. Our model of clinical mentorship is aimed to improve the quality of MNH care that is developed and implemented by taking into consideration the low resources and remote conditions of facilities in the DRC. For example, the clinical mentors are selected locally from the two provinces participating in this project. This takes into account the following factors: budget, knowledge of the area, sustainability, and feasibility as to whether the program can be scaled. This clinical mentoring program not only focuses specifically on the quality of human resources but also addresses other determinants of quality of care. Essential equipment and consumables are regularly provided through the UNICEF and World Bank support schemes, and the facilities included in this project are receiving equipment upgrades, although the upgrade amounts allocated by the World Bank’s PBF project may not be sufficient for labor, delivery, and postpartum rooms. Assessments of facility conditions and needs determines the availability of equipment, drugs, supplies, and training and is collecting information on current activities and performance during the baseline surveys and implementation phase of the clinical mentorship program [7]. We are strategically balancing facilities across arms of the World Bank’s PBF clinical trial, as we assume PBF facilities may have better equipment, drugs, and supplies. This also allows us to assess whether the effectiveness of the clinical mentorship intervention is impacted by improvements to facility conditions (e.g., PBF versus non-PBF facilities, etc.)

This project is a joint collaborative effort between UNICEF, the DRC Ministry of Health, Tulane University, the Provincial Divisions of Health in Kwango and Kwilu, DRC, and eight of their affiliated health zones. Success for the implementation of the project is dependent upon coordination, supervision, and monitoring activities between all parties. The monitoring & evaluation component that Tulane University is performing is testing the effectiveness of this model of clinical mentoring to improve the quality of care to mothers and their newborns in the health facilities of the eight health zones being targeted in the two provinces. The evidence generated from this project will be presented during a national workshop aiming at disseminating the findings and organizing the dialogue among key stakeholders. Findings from this study may lead to the development of a national strategy to improve the quality of MNH care through continued education and capacity building, as well as improvements to the collection of key MNH indicators to monitor activity of care providers. Additionally, this model may be scaled and replicated both nationally and internationally. This program is expected to generate some of the first scientific evidence on the effectiveness of clinical mentoring to improve maternal and neonatal health care and outcomes. If the intervention is confirmed to be effective, it will demonstrate the potential for the adaptation of this model of clinical mentoring program to a new context and promote future research to assess program scale and dissemination.

## Data Availability

The baseline survey has been completed and published [7]. All data baseline and endline collection tools/questionnaires for the surveys are available to public access [23].

## Abbreviations

DRC: The Democratic Republic of the Congo
EMEN: Every Mother Every Newborn
EmONC: Emergency obstetric and neonatal care
GRH: General reference hospital
HC: Health center
LMIC: Low- and middle-income countries
MNH: Maternal and newborn health
MOH: Ministry of Health
ODK: Open Data Kit
UNICEF: The United Nations International Children’s Emergency Fund
WHO: World Health Organization
